# Nucleocapsid Assembly of Baculoviruses

**DOI:** 10.3390/v11070595

**Published:** 2019-07-01

**Authors:** Shuling Zhao, Guanghui He, Yiheng Yang, Changyong Liang

**Affiliations:** 1 College of Bioscience and Biotechnology, Yangzhou University, Yangzhou 225009, China; 2 Joint International Research Laboratory of Agriculture & Agri-Product Safety, Yangzhou University, Yangzhou 225009, China

**Keywords:** nucleocapsid assembly, baculovirus, preformed capsid, DNA packaging, capsid proteins

## Abstract

The baculovirus nucleocapsid is formed through a rod-like capsid encapsulating a genomic DNA molecule of 80~180 kbp. The viral capsid is a large oligomer composed of many copies of various protein subunits. The assembly of viral capsids is a complex oligomerization process. The timing of expression of nucleocapsid-related proteins, transport pathways, and their interactions can affect the assembly process of preformed capsids. In addition, the selection of viral DNA and the injection of the viral genome into empty capsids are the critical steps in nucleocapsid assembly. This paper reviews the replication and recombination of baculovirus DNA, expression and transport of capsid proteins, formation of preformed capsids, DNA encapsulation, and nucleocapsid formation. This review will provide a basis for further study of the nucleocapsid assembly mechanism of baculovirus.

## 1. Introduction

Baculoviridae consists of a large family of circular dsDNA viruses with DNA molecules sized from 80 to 180 kbp that are encapsulated in enveloped rod-like capsids [1,2]. Baculoviruses are insect-specific pathogens that have been extensively applied for insect control [3]. A baculovirus generates budded virions (BVs) and occlusion-derived virions (ODVs), which are two phenotypes of virions in its life cycle. Although BVs and ODVs contain the same nucleocapsid, they perform different functions in virus infection. Nucleocapsids bud from the plasma membrane to obtain the envelopes with fusion proteins F or GP64 to generate BVs. BVs are able to launch successful systematic infection through entering into many cells and tissues mediated by the fusion proteins within a host larva [4,5]. Nucleocapsids are coated by their envelopes containing a series of *per os* infectivity factors from the host cell nuclear membrane to form ODVs that are further embedded in paracrystalline polyhedrin (Polh) structures to form occlusion bodies (OBs). Liquefied host insects release OBs into the environment to cause epidemics among host larvae [6]. Since the nucleocapsids are identical between BVs and ODVs [6], the proper assembly of the nucleocapsid is essential for the production of BVs and ODVs, and also for the infectivity of the baculovirus. With all of this in mind, revealing the mechanism of nucleocapsid assembly in baculoviruses is important in order to help scientists to understand baculovirus transmission.

## 2. Assembly of Nucleocapsids in Viruses

Virus nucleocapsid assembly is generally divided into two steps: production of capsid proteins and genome package. The assembly mechanism of viral nucleocapsids is very complex and varies between viruses, but most viruses generally adopt two main strategies. For most DNA and RNA viruses with genome sizes less than 20 kb, such as simian virus 40 (a small DNA virus) [7] and tobacco mosaic virus (a famous RNA virus) [8], the energy-independent system is used, where the nucleocapsid is assembled around the genome by free capsid proteins or subunits. On the contrary, large viruses tend to use the energy-dependent system, where the empty capsids are assembled, and then the genomic nucleic acids are recognized and pumped into the preformed capsids by ATP-driven motors. This process has been well studied in bacteriophage lambda [9] and herpes viruses [10]. Baculoviruses appear to assemble their nucleocapsids using the latter strategy.

## 3. Replication and Processing of Baculovirus Genomes

Baculovirus DNA replication is carried out in the virogenic stroma (VS), a specific region in the host cell nucleus. A baculovirus replicates its genome by rolling circle replication, which is a strand displacement replication occurring on circular dsDNA [11,12]. This type of replication can produce numerous copies of the genome in a short time. Additionally, baculovirus replication leads to a high recombination status [13,14]. Recombination-dependent replication plays an essential role in the replication cycle of many dsDNA viruses, especially DNA viruses with large genomes. Baculoviruses produce high molecular weight DNA with an apparently branched structure [15]. A sequence alignment revealed that all baculoviruses encode a gene (alkaline nuclease) homologous to the lambda *red* α exonuclease in bacteriophages, which is a typical exonuclease in the Red recombination system [16]. Alkaline nuclease interacts with a virus-encoded single-strand DNA binding protein LEF-3 to form a complex that is involved in DNA recombination [17,18].

The intermediates in recombination-dependent replication are complex branched structures that should be processed into unit-length cyclic dsDNA genomes before nucleocapsid assembly. VLF-1, a member of the lambda integrase family [19], may play a role in the genome processing. VLF-1 exhibits a structure-dependent binding activity to DNA molecules, with the highest binding activity to the recombination intermediate with a cruciform DNA structure [20]. However, the processing mechanism is unclear. In addition to VLF-1, the host recombination and repair system is likely involved in viral DNA processing, which has been identified in a lambda phage [21].

## 4. Expression and Transport of Structural Proteins of Baculovirus Nucleocapsid

VP39 (the major capsid protein) [22,23] and P6.9 [24], the DNA binding protein that condenses the viral genome to form a nucleocapsid core, were identified as components of baculovirus nucleocapsid in the 1980s. With the development of technology, especially proteomics [25,26], an increasing number of structural proteins have been identified, including 38K [25,26,27], VP1054 [26,28], Ac53 [29], VP91 [30], VLF-1 [26], BV/ODV-C42 [25,26], P78/83 [25,26], BV/ODV-EC27 [26,31], 49K [26,31], VP80 [25,26,32], and so on. Almost all of the genes encoding these structural proteins are conserved in all baculoviruses and are essential to virus proliferation. The most probable reason is that without any of these structural proteins, the nucleocapsid will be damaged and the virus will lose its infectivity. 

Interestingly, all structural genes appear to be late transcribed by a virus-encoded RNA polymerase that is resistant to α-amanitin [6]. The separation between early and late transcription is the onset of DNA replication. The mechanism of the coordination and consistency between structural protein expression and viral genome replication is unclear. This may be related to the balance between genome replication and virus nucleocapsid formation. If structural genes are early genes, they will be expressed before the replication of the viral genome and then viral genomic DNAs are packaged into viral nucleocapsids, which leads to few genomic DNAs being used as templates for DNA replication. In this case, the replication of genomic DNA is disrupted, which is not conducive to the reproduction of viruses [6]. It is thought that two major features of newly synthesized DNA are beneficial to late transcription. The first feature is that newly synthesized (naked) DNA may promote the activation of late promoters until p6.9 or other DNA-binding proteins accumulate to the level at which viral DNA is condensed and transcription is suppressed. Another major feature is that in the process of lagging strand synthesis, the synthesis of Okazaki fragments produces a large number of nicks and RNA-DNA junctions. It is suggested that the unligated junctions of Okazaki fragments may be used as short-term enhancers of late transcription. Once DNA replication is completed, the loading sites of late gene activators can be eliminated by removing RNA primers and ligating lagging stands, thus terminating late transcription [6]. Therefore, the coordinated relationship between structural gene expression and genome replication may be as follows.

(1) When viral genomic DNA begins to be replicated, structural proteins begin to be expressed. At first, the number of genomic DNAs and structural proteins are small, and only small quantities of nucleocapsids are formed in the VS. In this state, the nucleocapsids are more easily transported outside the nucleus and budded from the cell membrane to produce BVs.

(2) When the number of viral genomic DNAs is increasing and the expression of structural proteins is increasing, more and more nucleocapsids are assembled, and nucleocapsids begin to arrange in bundles. In this case, it becomes more difficult for the nucleocapsid to be transported outside the nucleus. Then the nuclear membrane invades and envelopes the nucleocapsids to form ODVs with single or multiple particle(s).

(3) When the viral genome stops replicating, and the nucleocapsid assembly is finished, the unpackaged genome DNA is coated by P6.9 and the expression of structural proteins is shut off. In this case, the very late genes are easily overexpressed in large quantities. A large number of P10 and polyhedrin proteins are produced, and finally ODVs are embedded to form polyhedra.

Since nucleocapsid assembly is performed at the VS in the host cell nucleus, all structural proteins of nucleocapsids need to be transported to the nucleus, and then aggregate at the VS. The exact mechanism of nucleocapsid protein transport is unclear. It is suggested that VP1054 is responsible for the correct localization of capsid proteins (including VP39, P78/83, and BV/ODV-C42) in the nucleocapsid assembly site by regulating the delivery of nucleocapsid proteins [28]. Additionally, BV/ODV-C42 is capable of transporting P78/83 to cell nuclei. It is hypothesized that BV/ODV-C42 interacts with P78/83 to form a complex and facilitates transportation from the cytoplasm to the nucleus in insect cell infected with the virus [33].

## 5. Formation of the Preformed Capsid

**Figure 1:**
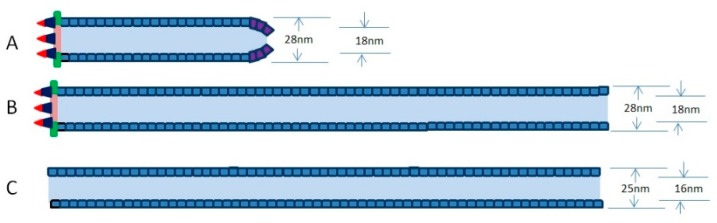
Simplified schemes of preformed capsid and tubular structure in cells infected with viable viruses (**A**) or inviable viruses (**B**,**C**). (**A**) A preformed capsid of nearly full length containing an apical cap, a cylindrical sheath, and a basal structure. (**B**) A preformed capsid grows longer and longer as a long tube when they are not packaged into viral genomes in time. (**C**) Excess VP39 proteins tend to self-assemble into the long tubular structure. In (**A**,**B**), the tubes are similar to W-tube (outer diameter of 28 nm and inner diameter of 18 nm), and the tube in (**C**) is similar to an N-tube (outer diameter of 25 nm and inner diameter of 16 nm).

Baculovirus nucleocapsid has a cylindrical sheath with an apical cap and a basal structure. When capsid proteins are transported to the VS, empty capsids will be assembled. The preformed capsids of nearly full length appear to begin with the assembly of the capsid sheath on a basal structure in the pockets of the VS [34]. The main component of the preformed capsid is VP39, which is the richest protein in viral particles [26]. Interestingly, a lot of long empty tubular structures were detected within the matrix of the VS or accumulated at the edge of the nucleus in the insect cells when a variety of different individual viral genes were deleted from the viral genome, including *ac53* [29], *38k* [27], *pk1* [1,35], *vp1054* [36], *bv/odv-c42* [31], *p49* [31], *bv/odv-ec27* [31], *vlf-1* [37], and *p6.9* [26], most of which are viral genes involved in nucleocapsid formation. It is unclear why the preformed capsid in cells transfected with these mutant viruses is not an empty capsid of suitable length, but an aberrant long tubular structure. The reasons may be as follows. (1) The normal empty capsids of nearly full length containing the apical cap, the cylindrical sheath, and the basal structure are able to be assembled in cells infected with replicative viable baculovirus (Figure 1A). (2) The normal empty capsids tend to grow longer and longer when they are not packaged into viral genomes in time (Figure 1B). (3) If the basal structure fails to be formed, and excess VP39 tends to self-assemble the long tubular structure (Figure 1C). 

The basal structure may consist of VLF-1 [37,38,39], BV/ODV-C42 [40], P78/83 [40], BV/ODV-EC27 [41], and other proteins. VLF-1 is a structural protein that is detected in two types of virions (BV and ODV) [42] and localizes to the ends of nucleocapsid [37]. P78/83 is a Wiskott-Aldrich syndrome protein (WASP)-like protein that activates Arp2/3 to drive the polymerization of G-actin into F-actin. The actin polymerization is the main driving force of nucleocapsid movement in cells [43,44]. However, P78/83 is not able to enter into the cell nucleus without the aid of BV/ODV-C42, which contains a nuclear localization signal [33,45]. The interaction of BV/ODV-EC27 with BV/ODV-C42 is detected in yeast two-hybrid assay, so BV/ODV-EC27 may be one of components of the basal structure [41]. VP39 is the only detectable component of the cylindrical sheath [31]. 38K, VP1054, Ac53, VP91, 49K, and VP80 are also classified as minor capsid proteins, but the localizations of these proteins in nucleocapsids are unclear. In cells transfected with baculovirus genome with *49k* gene deletion, some nucleocapsids appeared to be fully formed, but were unenveloped in the nucleus [46]. As such, the 49K may localize to the cap of nucleocapsids, because association of nucleocapsids with de novo envelopes is apparently mediated through the cap structure [34].

It has been suggested that VP39 self-assembles to form preformed capsid in host cells infected with baculovirus [31]. Recent report showed that *Helicoverpa armigera nucleopolyhedrovirus* (an *Alphabaculovirus*) VP39 expressed in *Escherichia. coli* self-assembled into long tubular structures in vitro. These long tubular structures contained two tube types, one was an N-tube (25 nm outer diameter and 16 nm inner diameter), and the other was a W-tube (28 nm outer diameter and 18 nm inner diameter) [47]. In *Spodoptera litura granulosis virus* (SpliGV, a *Betabaculovirus*), the cylindrical sheath of the empty nucleocapsid was detected to be similar to a W-tube [48]. The empty nucleocapsids of SpliGV extracted from granuloses are very similar to the normal nucleocapsid and contain the apical cap, the cylindrical sheath, and the basal structure [48]. Interestingly, the N-tubes are about 90% of the in vitro synthesis system, and the W-tubes are about 10% in vitro synthesis system, indicating that N-tubes are more easily formed in vitro [47]. As such, the assembly of the capsid sheath may need the help of other proteins in vivo. In our opinion, the capsid sheath containing the basal structure may be assembled according to the W-tube style (Figure 1B), while the capsid sheath lacking the basal structure may tend to be assembled according to the N-tube style in vivo (Figure 1C). Further investigation is required for a definitive confirmation.

## 6. DNA Packaging and Nucleocapsid Formation

Once viral DNA begins to replicate and its concentration becomes higher and higher, it triggers the viral DNA to be packaged into preformed capsid. DNA packaging may be coordinated with genome production. It has been suggested that the proteins that influence the replication of genome and DNA condensation may also influence the assembly of nucleocapsids. The knockout of DBP [49] or P6.9 [50] causes the empty tubular structure to be produced. P6.9 encoded by virus is a basic DNA-binding protein with only 50 aa residues [51] and is abundant in nucleocapsids [26]. Small basic proteins are able to condense genomic DNA and aid the packaging of DNA into capsids in DNA viruses. Host histones are frequently used for DNA packaging in some DNA viruses [52,53,54]. Interestingly, the phosphorylation status of P6.9 is very important to nucleocapsid assembly and viral gene transcription [55,56,57]. It has been reported that newly synthesized P6.9 is transiently phosphorylated [55], and hyperphosphorylation of P6.9 has been reported to be involved in viral very late gene hyperexpression [57]. However, the dephosphorylation of P6.9 plays an important role in viral genome packaging [24,58,59]. A recent report showed that 38K mediates the dephosphorylation of P6.9, which is essential to nucleocapsid assembly [60], suggesting that genomic DNA coated with dephosphorylated P6.9 is allowed to be condensed and packaged into the nucleocapsid. 

**Figure 2:**
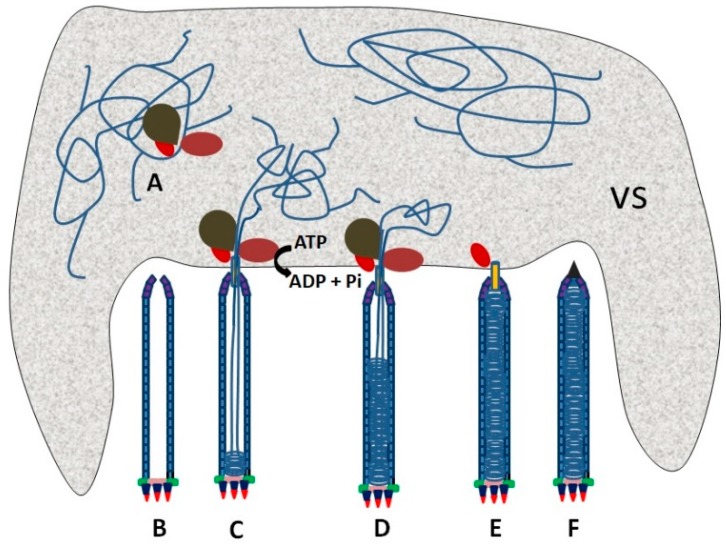
Theoretical schematic of the genome packaging in a baculovirus. (**A**) A *cis*-acting element is recognized by a packaging protein complex. (**B**) A preformed capsid is assembled and waits for docking of the viral genome onto it though the packaging complex. (**C**,**D**) The viral genome is packaged through a portal at the apical cap of the preformed capsid with the aid of ATP hydrolysis. (**E**) The packaging complex is released after a genome DNA with one unit length is packaged. (**F**) The portal of a capsid is blocked with structure protein(s) to form mature nucleocapsid. The shaded area is the virogenic stroma (VS).

In phages and some large viruses, genomic DNA is packaged into a preformed capsid by an ATP-driven motor that includes a portal protein and terminase-type enzyme(s) or other translocases. The portal protein acts as a channel for the injection of DNA, and the translocases pump the DNA into the preformed capsid [61]. This mechanism of packaging genomic DNA may also be used in baculoviruses. Combining the early microscopy observation of *Autographa californica multiple nucleopolyhedrovirus* nucleocapsid morphogenesis [34] with other knowledge of nucleocapsid assembly [6,61], we propose a model of baculovirus DNA packaging. In this model, DNA packaging may be generally divided into three steps. (1) The viral genome is docked onto the portal situated at the cap of a preformed capsid by packaging proteins (Figure 2A,B). (2) The genome is pumped into the preformed capsid through the portal by a packaging motor with the help of ATP hydrolysis (Figure 2C,D). (3) Packaging proteins are released from the nucleocapsid when the genome is fully packaged. The portal of the capsid is blocked with structure protein(s) to form mature nucleocapsid (Figure 2E,F).

In order to ensure that only the viral genome is packaged, recognition of a specific sequence in the viral genome is essential for nucleocapsid assembly (Figure 2A). It is suggested that VP1054 may be involved in DNA recognition. VP1054 is similar to PURα family proteins and is able to bind to GGN-rich sequences [36]. A GGN-rich region in *p78/83* was detected to interact with VP1054 [36]. Moreover, VP1054 was able to interact with the capsid protein 38K that catalyzed the dephosphorylation of P6.9 in genome packaging [62]. Interestingly, a *cis*-acting element named NAE containing eight conserved A/T-rich regions was identified in the *ac83* coding region and is essential to DNA packaging [2]. Homologs of NAE are detected only in alphabaculoviruses. Another essential *cis*-acting element conserved in alphabaculoviruses is found in the *ac152* coding region [63]. However, it is unknown which protein(s) can recognize those *cis*-acting elements. 

To date, the composition of the baculovirus motor complex is unclear. Ac66 may act as a candidate for a motor protein. Ac66 is related to some motor proteins [6]. However, the function of Ac66 in nucleocapsid assembly still needs to be explored. Additionally, actin plays an important role in DNA packaging. Structure components of nucleocapsid are able to interact with actin in the infected cell nucleus, indicating that actin is transported from the cytoplasm into the nucleus [64]. Empty tubular structures were detected in cells when actin polymerization was blocked [65], suggesting that actin is involved in genome packaging into preformed capsids.

## 7. Outstanding Question and Conclusions

An outstanding question concerns the parameters that regulate the length of the nucleocapsid to fit the size of the encapsulated DNA molecule. It has been reported that the length of capsids may be resilient to genome size [66]. It is unknown whether the lengths of preformed capsids could be changed according to the size of the genome. In addition, it is also possible that a preformed capsid of a fixed length could be elongated in DNA packaging according to the size of the genome. A lot of aberrant long electron-lucent tubular structures are frequently observed in cells infected with gene-deleted baculoviruses, suggesting that the nucleocapsid assembly of baculoviruses is very easy to disturb. Recently, an increasing number of nucleocapsid proteins and *cis*-acting elements were identified in order to expand our knowledge of baculovirus nucleocapsid assembly. Here, we made an attempt to review baculovirus nucleocapsid assembly. We believe that the mechanism of nucleocapsid assembly will aid us to better understand the life cycle of baculoviruses.
